# The Diagnosis and Clinical Significance of Paragangliomas in Unusual Locations

**DOI:** 10.3390/jcm7090280

**Published:** 2018-09-13

**Authors:** Sylvia L. Asa, Shereen Ezzat, Ozgur Mete

**Affiliations:** 1 Department of Pathology, University Health Network, Toronto, ON M5G 2C4, Canada; pathlady01@gmail.com; 2 Endocrine Oncology Site, Princess Margaret Cancer Center, Toronto, ON M5G 2MG, Canada; shereen.ezzat@uhn.ca; 3 Department of Laboratory Medicine and Pathobiology, University of Toronto, Toronto, ON M5S 1A8, Canada; 4 Department of Medicine, Division of Endocrinology, University Health Network, Toronto, ON M5G 2C4, Canada; 5 Department of Medicine, University of Toronto, Toronto, ON M5S 1A8, Canada

**Keywords:** pheochromocytoma, paraganglioma, genetic susceptibility, SDHB, metastatic paraganglioma, catecholamines

## Abstract

Paragangliomas are neuroendocrine neoplasms, derived from paraganglia of the sympathetic and parasympathetic nervous systems. They are most commonly identified in the head and neck, being most frequent in the carotid body, followed by jugulotympanic paraganglia, vagal nerve and ganglion nodosum, as well as laryngeal paraganglia. Abdominal sites include the well-known urinary bladder tumors that originate in the Organ of Zuckerkandl. However, other unusual sites of origin include peri-adrenal, para-aortic, inter-aortocaval, and paracaval retroperitoneal sites, as well as tumors in organs where they may not be expected in the differential diagnosis of neuroendocrine neoplasms, such as thyroid, parathyroid, pituitary, gut, pancreas, liver, mesentery, lung, heart and mediastinum. The distinction of these lesions from epithelial neuroendocrine neoplasms is critical for several reasons. Firstly, the determination of clinical and biochemical features is different from that used for epithelial neuroendocrine tumors. Secondly, the genetic implications are different, since paragangliomas/pheochromocytomas have the highest rate of germline susceptibility at almost 40%. Finally, the characterization of metastatic disease is unique in these highly syndromic lesions. In this review, we summarize updated concepts by outlining the spectrum of anatomic locations of paragangliomas, the importance of morphology in establishing the correct diagnosis, the clinical implications for management, and the impact of genetics on the distinction between multifocal primary tumors compared with malignant disease.

## 1. Distribution and Localization of Paragangliomas

**Figure 1:**
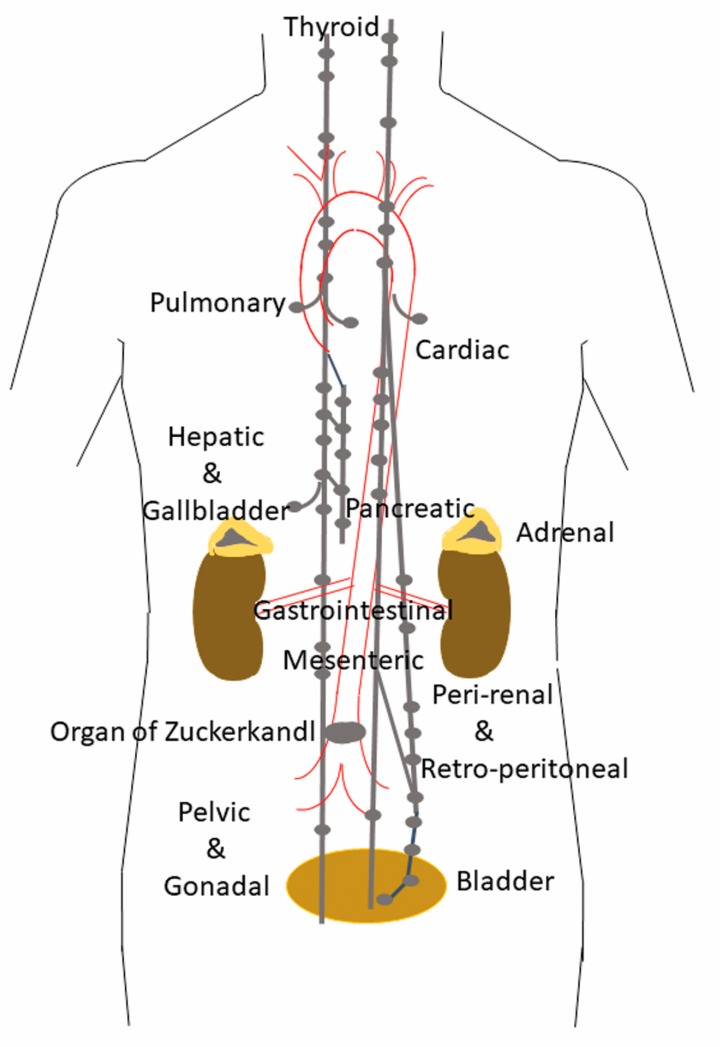
Location of paraganglia. This figure illustrates the location of normal paraganglia in the neck, thorax and abdomen. Locations in the head are not shown.

Paragangliomas (PGLs) are non-epithelial neuroendocrine neoplasms (NENs) [1] that derive from paraganglia, which are seen in close association with components of the sympathetic and parasympathetic nervous systems [2,3,4,5] They can arise in any location where paraganglia normally reside in adult tissues (Figure 1) or during embryonic development [6].

Parasympathetic paraganglia are distributed along the cranial and thoracic branches of the glossopharyngeal and vagus nerve [2]. The commonest site of tumor development involves the glossopharyngeal nerve, in the carotid bodies, which are located immediately above the bifurcation of the carotid arteries [3,4,7,8]. The second most common location is the middle ear, which contains multiple jugular and tympanic paraganglia that give rise to jugulotympanic paraganglia known as “glomus jugulare” and “glomus tympanicum”. This region is innervated by the auricular branch of the vagus nerve (Arnold’s nerve), and the tympanic branch of the glossopharyngeal nerve (Jacobson’s nerve) [6]. Glomus jugulare tumors may be associated with either Arnold’s or Jacobson’s nerve, whereas glomus tympanicum tumors are almost always associated with Jacobson’s nerve [6,9]. Tumors of the vagus nerve also include the laryngeal paraganglia, and the subclavian and aorticopulmonary or cardioaortic paraganglia near the bases of the great vessels or involving the interatrial septum of the heart.

Sympathetic paraganglia are located predominantly in the abdomen [2], in chains that run through the prevertebral and paravertebral connective tissue, as well as along the inferior hypogastric plexuses adjacent to the urogenital organs, and in the wall of the urinary bladder; the largest ones represent the bilateral adrenal medullas and the organ of Zuckerkandl. Interestingly, apart from the adrenal medulla, the distribution of these paraganglia is nearly identical to that of lymph nodes and the discovery of the organ of Zuckerlandl was initially reported by Emil Zuckerkandl to his mentor, Alfred Kohn, as an unusual lymph node [9]. This feature often results in imaging studies that are not unlike what is seen in lymphoproliferative disorders.

Because of this widespread distribution of paraganglia, paragangliomas can occur at virtually all locations in the body except within the brain and in bone, and although they have been reported in the extremities [10], these are exceptional curiosities. Given the normal distribution of paraganglia associated with components of the autonomous nervous system, it should be no surprise that paragangliomas can occur in the gallbladder [11,12], and the liver [8,13,14], where they are likely to arise from small abdominal vagus nerve branches. Paraganglia are present and can give rise to paragangliomas in various sites including the orbit, mandible, paranasal sinuses and sellar region [8,15,16,17,18,19,20,21,22,23], adjacent to or within the thyroid gland [8,24,25,26,27,28,29], in the parathyroid [30], in the mediastinum [8,31,32,33], within the lungs and heart [8,34,35,36,37,38], gut [8], and pancreas [8,39,40] and in the mesentery [8,41,42].

**Figure 2:**
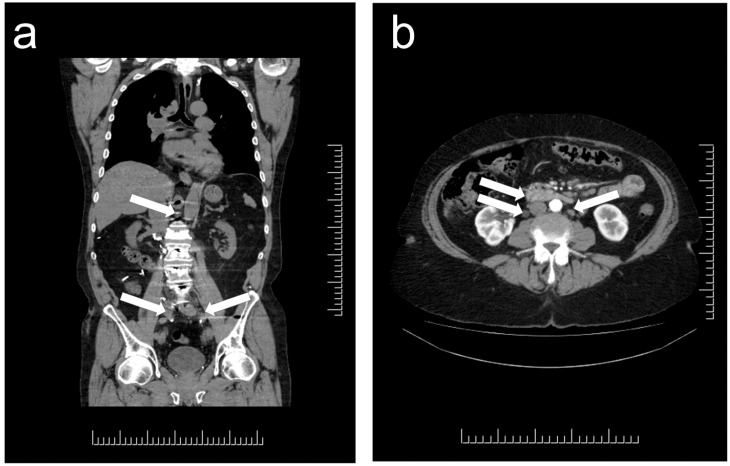
Localization of paragangliomas on Computed Tomography (CT) imaging. Multifocal paraganglia in patients with genetic predisposition to paraganglioma syndrome. (**a**) There are multiple small lesions all along the para-aortic channel (arrows), each representing a small paraganglioma in this patient with germline predisposition and multiple tumors, including multiple liver lesions. (**b**) In this patient with genetic predisposition to paraganglioma, there are multiple masses (arrows) that the radiology report identified as “multiple mesenteric lymph nodes, the largest one measures 5.1 × 1.9 cm, and there also sub-centimetric retroperitoneal lymph nodes”.

Paragangliomas that emerge in unusual anatomic locations can be a major source of confusion and diagnostic error. Imaging often reports the presence of paraaortic lymphadenopathy in patients with genetic predisposition to multifocal paragangliomas (Figure 2); this can be misdiagnosed on imaging as lymphoma or metastatic carcinoma in lymph nodes. Errors also occur with paragangliomas in liver and lung—patients with germline predisposition to paragangliomas can develop multiple primary tumors, and in these locations such tumors may be misclassified as “metastatic”. Furthermore, in some cases, these are misdiagnosed as metastatic neuroendocrine tumors (NETs). The 2017 World Health Organization (WHO) classification recognized that “Metastatic deposits should only be considered as such at sites where normal chromaffin tissue is not present, in order to avoid the misclassification of multicentric primary tumors as metastases” [43]. While liver and lung are included along with lymph nodes and bone, dispersed microscopic paraganglia associated with components of the autonomous nervous system in the liver and lung, make it imperative to also consider the possibility of primary paraganglioma in those locations. The possibility of metastatic disease can be favored when there is a known primary site and liver/lung involvement in a patient with no germline susceptibility to multifocal disease. In addition, paragangliomas are no longer classified as benign [44], since even without metastatic spread, multifocal or progressive disease can have significant morbidity and mortality. More important is to document the size, location and mutation status of a lesion to determine its likelihood of aggressive behavior [45,46].

## 2. Morphologic Diagnosis of Paragangliomas

**Figure 3:**
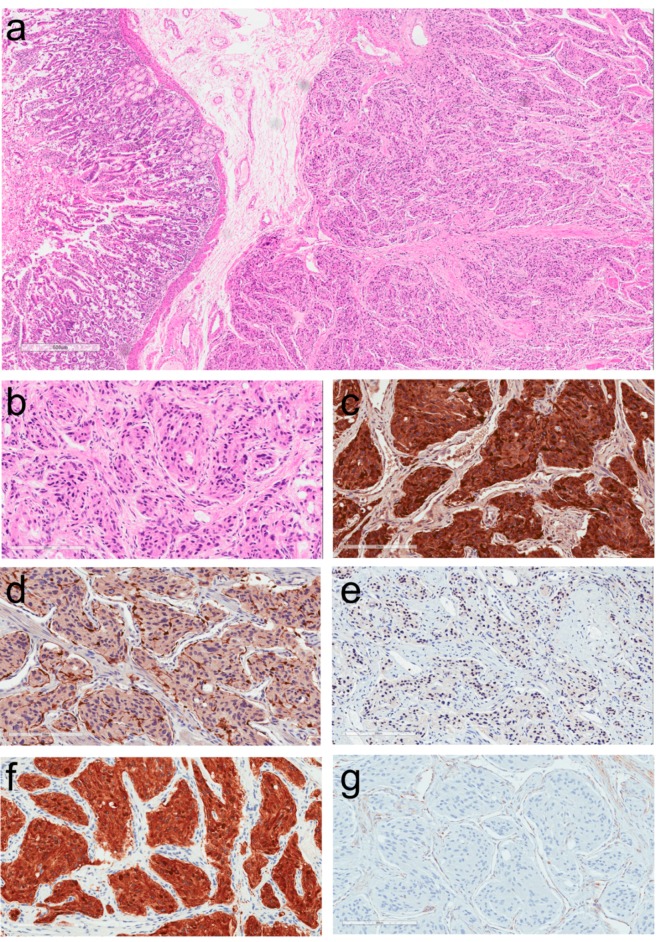
Histology and immunoprofile of a duodenal Succinate Dehydrogenase (SDH)-deficient paraganglioma. (**a**) This tumor is identified underlying duodenal mucosa and was classified as a small bowel neuroendocrine tumor. (**b**) The tumor cells are form small nests within a fibrovascular stroma. They are somewhat elongated and have moderate nuclear pleomorphism. (**c**) The tumor is strongly positive for chromogranin-A. (**d**) Sustentacular cells are highlighted by their intense reactivity for S100 protein. (**e**) There is nuclear staining for GATA-3 in tumor cells. (**f**) Strong cytoplasmic positivity for tyrosine hydroxylase confirms the diagnosis of paraganglioma. (**g**) This tumor is SDH-deficient as shown by lack of cytoplasmic granular reactivity for SDH subunit B (SDHB); note the positivity in stromal cells that serve as an internal control.

The histopathologic diagnosis of paraganglioma (PGL) can be simple when the lesion is in an expected location and when the patient presents with classical symptoms related to catecholamine excess. However, the diagnosis is often missed when they occur in unusual locations (Figure 3a). These tumors are usually composed of solid nests known as “zellballen” of round to oval or elongated cells with abundant granular amphophilic or basophilic cytoplasm, within a vascular stroma, but they may also have acidophilic cytoplasm (Figure 3b). There may be nuclear atypia but usually mitoses are scarce, and there is no necrosis. Immunohistochemistry plays a key role in confirming the diagnosis. However, many pathologists believe that the only stains required are chromogranin, synaptophysin and S100; the first two yield cytoplasmic positivity in tumor cells (Figure 3c) whereas the S100 stain, while it may stain tumor cells, provides a specific intense positivity that highlights sustentacular cells within these neoplasms (Figure 3d). The fallacy is that any type of NEN can stain for synaptophysin and chromogranin, and S100-positive sustentacular cells occur in NETs at many locations [47,48]. It is therefore important to identify other biomarkers that allow the distinction of PGL from NETs (epithelial NENs). In this regard, the importance of keratins cannot be overemphasized. Keratin stains are available in almost every pathology laboratory. The identification of a NEN that lacks keratin should prompt consideration of this diagnosis [49]. We recommend the use of pan-keratin antibodies, such as AE1/AE3, along with the CAM 5.2 antibody cocktail that highlights most NETs. While there are reports of unusual PGLs that may stain for keratins [13,14,37,40], the lack of keratin expression in a presumed NEN should raise the suspicion of PGL. In the authors’ experience, no PGLs other than the rare and unusual tumors classified as gangliocytic and cauda equine-type PGLs are positive for keratins.

**Figure 4:**
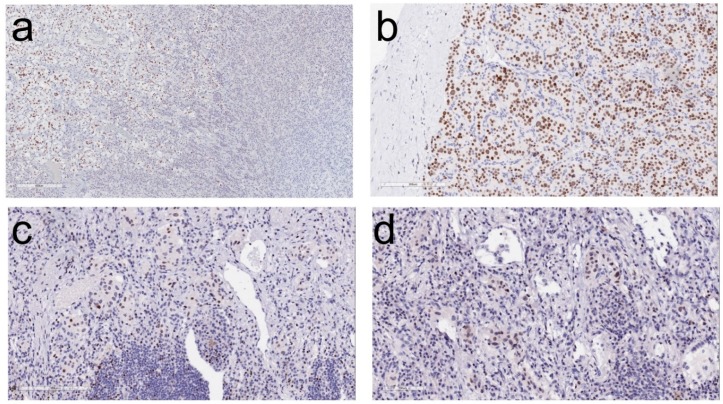
GATA-3 (GATA-binding factor 3) immunolocalization in adrenal medulla and paragangliomas. (**a**) In this section of normal adrenal, the medulla (left) stains for GATA-3 whereas the adrenal cortex (right) is completely negative. (**b**) A cardiac paraganglioma exhibits strong nuclear reactivity for GATA-3. (**c**,**d**) Lung paragangliomas have variable nuclear positivity for GATA-3.

When the diagnosis of PGL is suspected, it can be confirmed using stains for transcription factors. Most NETs express transcription factors that are specific to their site of origin [1,49] (Table 1). In contrast, PGLs are negative for these transcription factors and instead may express GATA-3 (Figure 3e), which is involved in the development of paraganglia [50]. A number of studies have confirmed this finding [51,52,53,54,55]. So and Epstein reported that 83% urinary bladder PGLs, 75% of PGLs from other sites, and 80% of metasatatic PGLs were positive for GATA-3; 79% of PGLs were positive for GATA-3 regardless of site of origin [53]. Miettinen et al. reported that GATA-3 is expressed in most pheochromocytomas (92%) and extra-adrenal PGLs (82%) [55]. In the authors’ experience, this biomarker is also very helpful in most PGLs and pheochromocytomas; examples of staining for GATA-3 are shown in Figure 4. This is a stain that is widely available and used for its application in identifying carcinomas of breast and urothelium; in the case of NETs, when positive, it points to either PGL or a parathyroid or pituitary [56] neoplasm that would likely be keratin-immunoreactive.

The importance of hormones and specific enzymes in the diagnosis of NETs is underestimated. In the case of PGLs, it is not feasible to stain for the hormones, dopamine, adrenaline and nor-adrenaline, however, there are antibodies to the various enzymes involved in catecholamine synthesis [4,57,58,59]. The rate limiting step in this enzymatic cascade involves tyrosine hydroxylase, the enzyme that converts L-tyrosine to L-DOPA (L-3,4-dihydroxyphenylalanine). Staining for tyrosine hydroxylase (Figure 3f) is, therefore, a convenient and reliable method to confirm the diagnosis of almost all PGLs and pheochromocytomas [60]. The main exception involves those arising from parasympathetic paraganglia that may be negative for this stain, given their high frequency of non-functionality. Staining for phenylethanolamine N-methyltransferase (PNMT) that converts norepinephrine to its terminal metabolite epinephrine can assist in distinguishing tumors that make epinephrine from those that do not and are negative for this final enzyme in catecholamine biosynthesis.

An interesting phenomenon that raises questions about the distinction between paragangliomas and epithelial NETs is the existence of rare “gangliocytic PGLs” and “cauda-equina type PGLs” that are positive for keratins [2]; while most of these have not been assessed with either GATA-3 or tyrosine hydroxylase immunohistochemistry (see below), an endobronchial gangliocytic PGL showed positivity for tyrosine hydroxylase expression [35]. Some authors consider these tumors to be PGLs with divergent epithelial differentiation, like ependymal differentiation, and similar to other non-epithelial tumors (e.g., epithelioid angiosarcoma) that display variable keratin expression. However, it remains to be determined whether these tumors should be classified as PGLs or as epithelial NETs.

## 3. Clinical Implications of the Diagnosis of Paraganglioma

The importance of the correct diagnosis and the distinction of PGL from NETs has a number of clinical implications.

First and foremost is the correlation of clinical symptoms with pathology and the provision of biomarkers for tumor surveillance. The clinical presentation with sweating, diarrhea, anxiety and intermittent hypertension can resemble the signs and symptoms of carcinoid syndrome. Patients diagnosed with NETs of the small bowel and abdomen are routinely diagnosed and followed with measurements of the urinary serotonin derivative 5-HIAA (5-hydroxyindoleacetic acid). If the clinical and pathological misdiagnosis is NET, this biomarker will be falsely normal. Patients with PGLs should instead be diagnosed and monitored with more specific measurements of catecholamines unique to their PGL. There is consensus that the biochemical testing is best aimed at measuring N-metabolites of catecholamines (methoxytyramine, normetanephrine and metanephrine) rather than the parent catecholamines themselves (dopamine, norepinephrine, and epinephrine) [58,61,62,63]. Despite the importance of routine biochemical testing for catecholamine derivatives, many practices have not yet adopted routine testing of these biomarkers especially for head and neck PGLs (e.g., carotid body paragangliomas). Most of these tumors may appear to be clinically non-functional but they can secrete dopamine, and/or noradrenaline [63,64]. While methoxytyramine is not routinely offered by most laboratories, the accurate determination of the catecholamine profile is crucial in the clinical management and surveillance of these patients. The risks of catecholamine excess are not trivial, as they include extreme lability of blood pressure, cardiac dysfunction, and acute vascular catastrophes.

The second issue is the need for genetic testing that is implied by this diagnosis. More than 40% of patients with these neoplasms carry germline mutations involving one of more than 20 genes [65,66,67,68]. The identification of germline disease has implications for the patient in interpreting disease progression as new lesions appear, and also for the family; the diagnosis of a PGL in a young patient can be expedited and complications prevented by screening of family members [34,38]. The major genetic alterations involve *RET* (rearranged during transfection) is the causative gene underlying multiple endocrine neoplasia (MEN) type 2A and 2B that are more often associated with pheochromocytomas), *VHL* that causes von Hippel–Lindau disease, *NF1* associated with neurofibromatosis type 1, and the *SDHB*, *SDHD*, and *SDHC* genes encoding subunits B, D, and C of the succinate dehydrogenase enzyme complex involved in the Krebs cycle. More rarely, germline mutations are identified in *SDHA* (succinate dehydrogenase subunit A), *TMEM127 (t*ransmembrane protein 127), *FH* (fumarate hydratase), *KIF1Bβ* (Kinesin-like protein), *MAX* (MYC-associated factor X), *HIF2A* *(*hypoxia-inducible factor 2 alpha) or *EPAS1*, *PHD1* (prolyl hydroxylase) (also known as *EGLN2*), *EGLN1* (formerly known as PHD2), *SDHAF1, SDHAF2* (the Succinate Dehydrogenase Assembly Factors), *BAP1* (BRCA1 associated protein-1), and *KMT2D* (Histone-lysine N-methyltransferase 2D; also known as MLL2) and *DNMT3A* (DNA cytosine-5-methyltransferase 3A) [68,69,70,71,72,73].

Pathologists can assist in triaging the diagnosis of genetic predisposition by applying immunohistochemistry. The use of antibodies to SDHB provides a screen for all *SDHx*-related disease [74], since any mutation involving one of the SDH subunit and the assembly factors results in destabilization of the protein complex and loss of immunoreactivity (Figure 3g). When negative, staining for SDHA (succinate dehydrogenase subunit A) can then be added to determine *SDHA*-related disease [75,76]. An antibody against *SDHD*-related pathogenesis has also been introduced [77], but the global experience is lacking for most recent antisera other than SDHB and SDHA. Similarly, staining for FH or 2-SC (2-succinyl cysteine) can be used to identify *FH*-related disease [74]. MAX antibody can also be used in the distinction of *MAX*-related pathogenesis [67,78], however, experience on the performance of this antibody is limited. *VHL*-related PGLs can be highlighted using carbonic anhydrase IX immunohistochemistry [79]; the specificity of this biomarker remains to be confirmed, as it may also reflect other alterations in the hypoxia pathway. When the patient has adrenal medullary disease, known as pheochromocytoma, the presence of associated adrenal medullary hyperplasia indicates that *RET* pathogenesis is most likely, since this phenomenon is typically found in patients with MEN2 [80]. However, recent evidence also suggested that adrenal medullary hyperplasia may also occur in patients with *TMEM127, MAX* and *SDHB* mutations [81,82,83,84].

## 4. Impact of Genotype-Phenotype Correlations in Paraganglioma

There is a strong genotype-phenotype correlation with respect to the tumor catecholamine profile, anatomic location, and risk of metastatic spread in PGLs.

**Figure 5:**
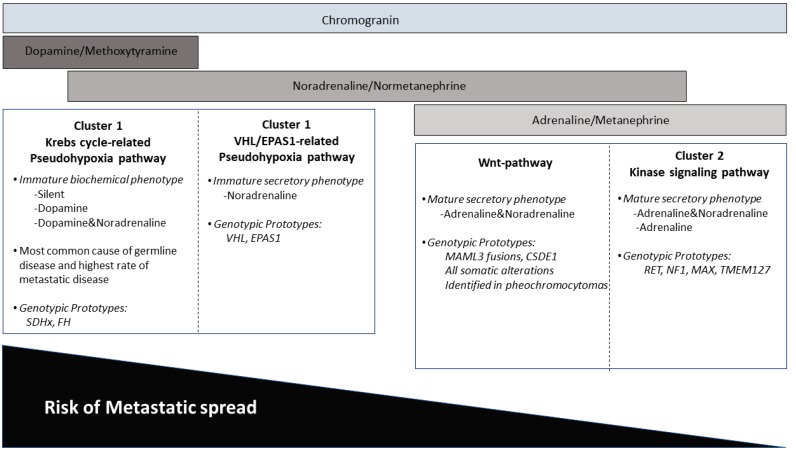
Biochemical and genetic clusters of paragangliomas.

The diagnosis of PGL requires biochemical assessment to determine the pattern of catecholamine production by the tumor. This biochemical profile can also provide insight into a familial syndrome, since each of the familial syndromes is associated with a distinctive functional profile. This has given rise to the concept of tumor genotype clusters (Figure 5). Therefore, biochemical information can provide guidance to testing for germline predisposition, as there is a strong genotype-biochemical phenotype correlation in these neoplasms [4,59,68]. PGLs or pheochromocytomas that are linked to a pseudohypoxic pathway (e.g., *SDHx*, *VHL*, *HIF2a, FH*, *MDH2*, *PHD1/EGLN2*, *PHD2/EGLN1*) are referred to as Cluster 1 disease, whereas tumors that are linked to a cluster rich in kinase receptor signaling pathways (e.g., *RET, TMEM127*, *MAX*, *NF1*, *KIF1Bβ*) are referred to as Cluster 2 disease. Dopaminergic and/or noradrenergic secretory profiles are characteristic features of functional tumors identified in Cluster 1, whereas adrenergic or mixed noradrenergic and adrenergic secretory phenotypes correlate with Cluster 2 disease. A subset of sporadic pheochromocytomas linked to wnt-altered pathway also shows mixed noradrenergic and adrenergic secretory phenotype [66,85].

The molecular cluster of a PGL may also impact the selection of imaging studies used for diagnosis and surveillance. Tumors with Cluster 1 profiles, especially those related to the pseudohypoxic pathway, express somatostatin receptors, therefore are well visualized with ^68^Ga-DOTATATE PET/CT and can also be localized with the less sensitive indium-labeled somatostatin scintigraphy. In contrast, tumors with Cluster 2 profiles can be imaged with ^18^F-DOPA PET/CT or Iobeguane, also known as metaiodobenzylguanidine (^123^I-MIBG), a radiolabeled molecule that is similar to noradrenaline. The United States Endocrine Society Task Force for Pheochromocytoma has proposed that ^18^F-FDG PET/CT should be used for assessment of metastatic pheochromocytoma and PGL [62]; however, recent evidence has suggested that the performance of ^68^Ga-DOTATATE PET/CT was superior to both ^18^F-FDG-PET/CT and ^18^F-DOPA PET/CT imaging modalities for detecting multifocal primary and metastatic disease [85,86,87].

The results of genetic testing also impact the likelihood of metastatic potential. In fact, while there are multiple pathological features that have been implicated in scoring systems to predict the likelihood of metastasis, including the PASS (Pheochromocytoma of the Adrenal gland Scaled Score) [88] and GAPP (Grading of Adrenal Pheochromocytoma and Paraganglioma) [89] scores, these were created without the confounding factor of germline predisposition. Indeed, it may be that prior to the WHO 2017 classification [43,44], some of the cases that were classified as “metastatic” or “malignant” were instead multifocal primary tumors. It is now known that malignant behaviour defined on the basis of metastatic spread. Large tumor size, extra-adrenal tumor location, catecholamine profile and *SDHB* mutation status are all considered important risk factors for metastatic behavior [2,45,46,68]. In contrast, the risk of biological aggression in sporadic disease (especially in pheochromocytoma) has been linked to a novel molecular pathway known as the wnt-altered pathway characterized by *MAML3* (Mastermind Like Transcriptional Coactivator 3) oncogene fusions along with other alterations including *ATRX* (Alpha Thalassemia/Mental Retardation Syndrome X-Linked) and *CSDE1* (Cold Shock Domain Containing E1) mutations [66]. The 5-year survival rate of individuals with metastatic pheochromocytomas and PGLs was reported to be 63% in a large meta-analysis [90]. Nevertheless, the concept of attributing metastatic spread to these tumors is now considered to be less critical than identifying the genetic mutation underlying the disease.

The identification of *SDHx-*related pathogenesis has also several clinical implications: (**i**) The increased risk of metastatic and multifocal primary disease, (**ii**) the possibility of synchronous neoplastic manifestations other than PGL or pheochromocytomas (e.g., gastrointestinal stromal tumor, renal cell carcinoma, pituitary neuroendocrine tumor, thyroid carcinoma, pancreatic neuroendocrine tumor, and adrenal cortical carcinoma) [91,92,93,94]. For this reason, affected individuals require lifelong careful clinical surveillance.

## 5. Conclusions

The clinical, biochemical, radiologic and morphological features of PGLs are all important features that are required to properly structure a management plan for patients with such tumors. Clarity is assisted by using a comprehensive pathology reporting module [60], that ensures collection and collation of the various components required for proper diagnosis. Awareness of the existence of PGLs in unusual locations and the likelihood of multifocal primary tumors in patients with germline genetic predisposition to this disease will reduce errors in diagnosis and provide more accurate data collection to allow progress in the understanding of these fascinating tumors.

## Figures and Tables

**Table 1:** Transcription Factors Distinguishing Various Neuroendocrine Neoplasms in the Differential Diagnosis of Paragangliomas in Unusual Locations.

Site	Transcription Factors Frequently Expressed
Pituitary	Pit-1, Tpit, SF-1, ER-alpha, GATA-3, GATA-2
Thyroid	PAX-8 *, TTF-1
Parathyroid	GATA-3, GCM-2
Lung	TTF-1
Stomach	CDX-2
Duodenum	ISL-1, PDX-1, CDX-2
Pancreas	PDX-1, ISL-1, CDX-2
Jejunum/Ileum	CDX-2
Appendix	CDX-2
Colon/Rectum	CDX-2
Paragangliomas	GATA-3

Pit-1: pituitary specific transcription factor 1; Tpit: T-box transcription factor; SF-1: Steroidogenic factor-1, ER-alpha: Estrogen receptor alpha, GATA-3: GATA-binding factor 3, GATA-2: GATA-binding factor -2; PAX8: Paired Box 8; TTF-1: Thyroid transcription factor-1; GCM-2: Glial cell missing 2; CDX-2: Caudal Type Homeobox 2; PDX-1: Pancreatic and Duodenal Homeobox-1; ISL-1: ISL LIM Homeobox 1. (*) Monoclonal.
